# *Wolbachia*-Induced Unidirectional Cytoplasmic Incompatibility and Speciation: Mainland-Island Model

**DOI:** 10.1371/journal.pone.0000701

**Published:** 2007-08-08

**Authors:** Arndt Telschow, Matthias Flor, Yutaka Kobayashi, Peter Hammerstein, John H. Werren

**Affiliations:** 1 Center for Ecological Research, Kyoto University, Kyoto, Japan; 2 Institute for Theoretical Biology, Humboldt University, Berlin, Germany; 3 Department of Biology, University of Rochester, Rochester, New York, United States of America; University of Sheffield, United Kingdom

## Abstract

Bacteria of the genus *Wolbachia* are among the most common endosymbionts in the world. In many insect species these bacteria induce a sperm-egg incompatibility between the gametes of infected males and uninfected females, commonly called unidirectional cytoplasmic incompatibility (CI). It is generally believed that unidirectional CI cannot promote speciation in hosts because infection differences between populations will be unstable and subsequent gene flow will eliminate genetic differences between diverging populations. In the present study we investigate this question theoretically in a mainland-island model with migration from mainland to island. Our analysis shows that (a) the infection polymorphism is stable below a critical migration rate, (b) an (initially) uninfected “island” can better maintain divergence at a selected locus (e.g. can adapt locally) in the presence of CI, and (c) unidirectional CI selects for premating isolation in (initially) uninfected island populations if they receive migration from a *Wolbachia*-infected mainland. Interestingly, premating isolation is most likely to evolve if levels of incompatibility are intermediate and if either the infection causes fecundity reductions or *Wolbachia* transmission is incomplete. This is because under these circumstances an infection pattern with an infected mainland and a mostly uninfected island can persist in the face of comparably high migration. We present analytical results for all three findings: (a) a lower estimation of the critical migration rate in the presence of local adaptation, (b) an analytical approximation for the gene flow reduction caused by unidirectional CI, and (c) a heuristic formula describing the invasion success of mutants at a mate preference locus. These findings generally suggest that *Wolbachia*-induced unidirectional CI can be a factor in divergence and speciation of hosts.

## Introduction

Reproductive parasites like intracellular bacteria *Wolbachia* manipulate the reproductive system of their hosts to their own benefit. Most commonly, *Wolbachia* cause sperm-egg incompatibilities, known as cytoplasmic incompatibility (see [1], [2] for reviews). In concert with geographic or genetic barriers to gene flow, such cytoplasmic incompatibility (CI) could promote the evolution of reproductive isolation, a crucial component in speciation [3]–[6]. *Wolbachia* are widely distributed in arthropods, with an estimate of 20% to 70% insect species infected with the bacteria [7]–[9]. Hence, addressing whether *Wolbachia* affects the speciation processes of its hosts is an important question. Here, we investigate theoretically the effect of unidirectional CI on speciation. Our modeling approach is in line with the theoretical literature on both speciation by reinforcement and the invasion of modifiers of mating (e.g. [10]–[13]).

Cytoplasmic incompatibility (CI) is a mating incompatibility induced by *Wolbachia* (see [14] for a review). It is called cytoplasmic because *Wolbachia* is transmitted from the mother through the cytoplasm of the egg to the offspring. There are two basic forms. Unidirectional CI involves one *Wolbachia* strain. If the father is infected with *Wolbachia* then matings with uninfected females (or females infected with a different strain) have a reduced number of surviving offspring in comparison to other possible matings. This is due to an incompatibility between egg and sperm [15]. Bidirectional CI is caused by two *Wolbachia* strains and can occur when mating partners are infected with different strains.

Cytoplasmic incompatibility has attracted attention as a possible mechanism for rapid speciation [1], [3], [4], [6], [16]–[18]. The basic idea is that CI reduces gene flow between populations, permitting genetic divergence and selecting for premating isolation. In the case of bidirectional CI there is both empirical and theoretical evidence supporting this view. Field studies show that many insect species harbor different strains of *Wolbachia*, often in different geographic regions [19]–[22]. Further, crossing experiments suggest that bidirectional CI is a major isolation factor between some strains and closely related species [4], [5], [17], [23]–[25]. Theoretically, it has been shown that two *Wolbachia* strains can stably coexist in parapatric host populations in the face of substantial migration [26], and that bidirectional CI reduces the gene flow of locally adapted alleles and selects for premating isolation even if the transmission of *Wolbachia* and the level of incompatibility are incomplete [6], [27], [28].

However, the view that *Wolbachia* are significant factors in arthropod speciation is controversial [3], [18], [29]–[32]. Common criticisms are that bidirectional CI (the mode that most obviously can promote reciprocal reductions in gene flow) will be uncommon in nature, that levels of CI are insufficient to allow genetic divergence, and that CI is not effective in promoting the evolution of premating isolation.

Unidirectional CI is likely to be more common in nature, since it requires that only one population be infected with *Wolbachia*. However, unidirectional CI is generally not believed to promote speciation in hosts because maintenance of infected and uninfected populations is expected to be unstable in the presence of migration. Therefore, CI differences would not persist and gene flow would eliminate differences between diverging populations. A prominent example for the instability of unidirectional CI is the rapid spread of *Wolbachia* in *Drosophila simulans* of California [33]. However, in other field studies mixed infections have been observed between populations of a species [5], [34], [35]. One example is the absence of *Wolbachia* in invasive populations of fire ants in North America, but presence of infections in source populations in South America [5]. The converse pattern is also found, presence of *Wolbachia* in globally distributed *Culex pipiens* but its absence in a smaller potential source population in South Africa [35]. Further, in mushroom feeding *Drosophila* unidirectional CI between a closely related infected and uninfected species may be a major factor of genetic isolation [5], [36]. Reinforcement of reproductive isolation appears to be occurring in the contact zone. These experimental studies indicate that further modeling of the dynamics of unidirectional CI and its possible role in promoting reproductive isolation is needed. The basic question is whether there are conditions under which unidirectional CI can create a stable infection pattern and whether this selects for genetic divergence and premating isolation. So far there is one theoretical study addressing this question. Flor et al. [37] demonstrated analytically that infected and uninfected parapatric host populations can stably coexist if migration is below a critical migration rate. The critical migration rate was shown to be positive if *Wolbachia* causes either a fecundity reduction in the host or its transmission is incomplete.

In the present study we investigate the role of *Wolbachia*-induced unidirectional CI on host speciation more generally. We first consider the conditions that permit maintenance of infection differences between an infected mainland and an initially uninfected island population in the presence of unidirectional migration and local adaptation. We then follow Telschow et al. [6] and combine models for the *Wolbachia* dynamics [27], [38] with a well-studied reinforcement model [13]. This new model allows us to investigate the effect of unidirectional CI on genetic divergence of the host. We consider selection acting on a small (initially uninfected) island population experiencing migration from a large (infected) mainland population. The model includes a mate preference locus, a male trait locus undergoing divergent selection in the two populations, and cytoplasmic incompatibility. In the present study we demonstrate that island populations with low *Wolbachia* infection frequencies are able to maintain local adaptation in the face of migration better than can island populations with an infection. When combined, locally adapted alleles and differences in infection are both stable at higher rates of migration than either would be alone. In addition the infection difference between mainland and island allows premating isolation to evolve more readily. The results suggest that, if recurrent peripheral populations occur, it is the ones that lose their *Wolbachia* that are more likely to diverge into new species, and that unidirectional CI can select for premating isolation by reinforcement of mate discrimination.

## Model

**Figure 1:**
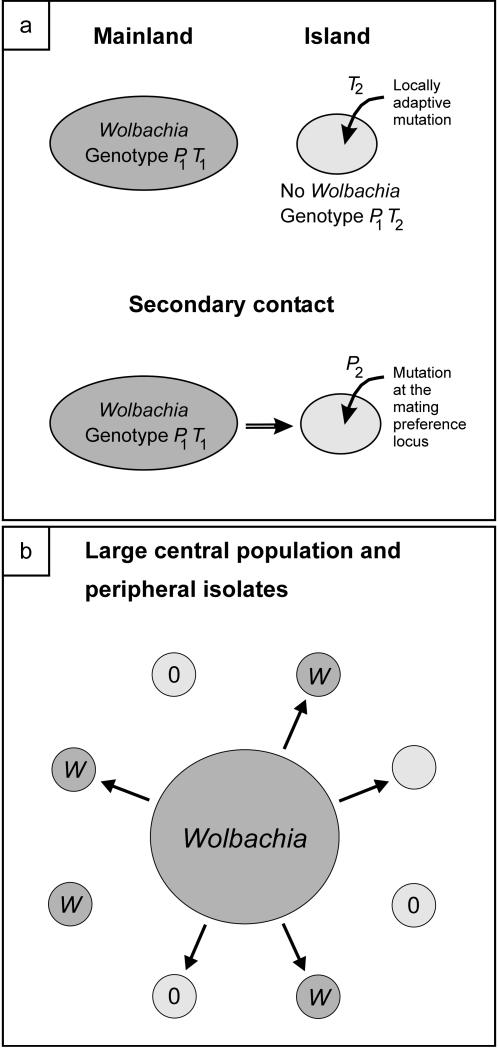
Model scenario. *Graph (a):* The ancestral situation is a large mainland population infected with *Wolbachia* and a small peripheral island population without *Wolbachia*. Further, the populations have diverged at a phenotypic trait locus *T*, with local adaptation in the island population of the *T*_2_ allele. After migration from mainland to island is restored (secondary contact), a new allele at the mating preference locus, *P*_2_, is introduced at low frequency in the island population. Females with *P*_2_ prefer *T*_2_ males relative to *T*_1_ males. We investigated the stability of infection differences between the populations, probability of local adaptation at the selected locus in the presence of migration, under what conditions mutants at the mate preference locus can invade and result in genetic divergence and prezygotic isolation. *Graph (b):* The mainland-island model analyzed in this article applies to a situation with a large central population and small peripheral isolates. The peripheral isolates might face migration from the infected mainland or not. Some of the isolates are infected with *Wolbachia* while others have lost the infection. Our results suggest that it is the ones that lose their *Wolbachia* that are more likely to diverge into new species.

Our model is similar to Dobzhansky's classical model of speciation by reinforcement (fig. 1, [39]). We assume that an ancestral host population has split into two populations, a large mainland and a small island. The scenario is analogous to a large central population with a small peripheral isolated population. The populations remain for some time in allopatry and diverge during that time at a locus which controls a male trait used in female mate choice. Further, the mainland is infected with *Wolbachia* but there is no infection on the island. After the establishment of these genetic differences the populations restore contact via migration from the mainland to the island (secondary contact). For low migration rates and if either *Wolbachia* transmission is incomplete or the infection reduces female fecundity, this infection pattern of infected mainland and (mostly) uninfected island is stable and, further, unidirectional CI acts as a postzygotic isolation mechanism. We first determine the stability of the infection difference between the populations, and then investigate whether postzygotic isolation selects for premating isolation and thus reinforces the genetic differences between the populations. To analyze when reinforcement takes place, we introduce mutants at a locus for female mating preference and study under which circumstances such mutants can invade, and whether this results in divergence at the preference locus.

For simplicity, we assume haploid sexual organisms, an assumption that often has been made for theoretical analyses involving multiple interacting loci (e.g. [13], [40]). Generations are discrete and non-overlapping. Individuals reproduce sexually with a primary sex ratio of 1∶1. The life cycle consists of four steps: migration, viability selection, sexual selection, and reproduction. It is assumed that the first three steps happen in the haploid phase of the organism. The diploid phase occurs during reproduction and ends with the production of the male and female gametes.

Organisms are characterized by their cyto-genotype. Individuals can be either infected with *Wolbachia* or not. Two nuclear loci are considered which recombine fully, a locus *P* for female mating preference, and a locus *T* for a male trait. Although we have not analyzed intermediate rates of recombination in this study it is worth noting that linkage between trait and preference locus will assist the spread of mutants at the preference locus and, further, increase the frequencies of the preferred male trait.

The life cycle starts with migration of the haploid organisms. Migration occurs only from the mainland to the island. Each generation a fraction *m* on the island is replaced by mainland organisms. After migration, viability selection takes place. We assume that selection on male trait alleles acts differently in mainland and island. On the mainland, *T*_1_ is favored and therefore fixed. On the island, however, *T*_2_-individuals have a (1+*s*)-times higher viability than *T*_1_-individuals. Therefore, there is differential selection for the trait on the island relative to the mainland.

Mating is nonrandom due to female mating preferences. We assume that females of genotype *P*_1_ and *P*_2_ have characteristic mating preference strengths, *a*_1_ and *a*_2_, respectively. In all simulations shown below, females with genotype *P*_1_ show no mating preference, i.e. *a*_1_ = 1, whereas *P*_2_ genotype females favor mating with *T*_2_ males, i.e. *a*_2_>1. Mating of *P*_1_ females is random and the frequency of mating with *T*_1_ and *T*_2_ males is proportional to the relative abundance of the two male traits. *P*_2_ females, however, mate *a*_2_-times more often with *T*_2_-males than *P*_1_ females do. The frequency of mating with the different male types is proportional to the relative abundance of the respective male trait weighed by the strength of mating preference. Normalization ensures the same overall number of matings for *P*_1_ and *P*_2_ genotype females (see Text S1 for a mathematical description). This structure using differential selection at a trait locus and a preference for that trait is similar to other modeling efforts of mate preference (e.g. [6], [13]).

After sexual selection the haploid individuals produce gametes that fuse to a diploid zygote. In this diploid phase some individuals suffer from *Wolbachia*-induced unidirectional CI. Following Fine [38], we describe the *Wolbachia* dynamic by three parameters: (a) the level of cytoplasmic incompatibility, *l*_CI_, defined as the fraction of offspring that die in matings between infected males and uninfected females, (b) the fecundity reduction, *f*, of infected females relative to uninfected females, and (c) the transmission rate, *t*, defined as the fraction of offspring which inherit the infection from their mother. Note that *Wolbachia* is transmitted only maternally through the cytoplasm of the egg.

The verbal description of the life cycle can be formalized. The corresponding mathematical model consists of a system of 8 coupled difference equations. A precise mathematical description is given in the supplementary material (Text S1). Table 1 summarizes the definitions of the parameters and symbols. In general, the model is too complex to be solved analytically. Therefore analytical results are only given for special cases and computer simulations were performed to analyze the general model (see [6] for details concerning the simulations).

**Table 1:** Definitions of the parameters and symbols used in the model.

Parameter	Description
*m*	migration rate
*l_CI_*	level of cytoplasmic incompatibility
*F*	fecundity reduction of infected females
*T*	transmission rate of Wolbachia
*S*	viability selection coefficient at locus *T*
*a*_1_; *a*_2_	mating preference strength coefficient
*T*_1_; *T*_2_	alleles at male trait locus *T*
*P*_1_;*P*_2_	alleles at mating preference locus *P*

Note that on the island individuals with genotype *T*_2_ have a (1+*s*)-times higher probability to survive than *T*_1_ individuals. Furher, females of genotype *P*_2_ mate *a*_2_ times more often with *T*_2_ males than with *T*_1_ males. In our simulations *P*_1_ females show no mating discrimination (*a*_1_ = 1).

## Results

Throughout the following sections we assume complete *Wolbachia*-transmission, i.e. *t* = 1. The case of incomplete transmission is considered in the supplementary material (Text S5).

### Stability of Postzygotic Isolation

In the first part of the results section we investigate under which circumstances the island remains at low infection frequencies in the face of migration from an infected mainland. In this situation there is an infection polymorphism between the infected mainland and the (mainly) uninfected island, and unidirectional CI causes postzygotic isolation between the two populations. We analyze the stability of this postzygotic isolation just after secondary contact and in the absence of sexual selection.

#### Critical Migration Rate

Previously, it was shown for both bidirectional CI and nuclear based mating incompatibilities that the stability of postzygotic isolation can be described in terms of a *critical migration rate* [6], [26]. It was shown that postzygotic isolation between two populations is stable if migration is below a critical value. However, if this critical migration rate is exceeded, postzygotic isolation gets inevitably lost. This is because selection is frequency dependent on the mating incompatibilities. Here, we follow Flor et al. [37] and use the term critical migration rate in the context of unidirectional CI and define the *critical migration rate* as the highest migration rate below which an infection polymorphism between an infected mainland and a (mainly) uninfected island can stably persist. The critical migration rate is a function of the parameters describing the *Wolbachia* dynamic as well as of the selection coefficient associated with the male trait and is denoted by *m_c_* = *m_c_*(*l*_CI_, *f*, *s*).

**Figure 2:**
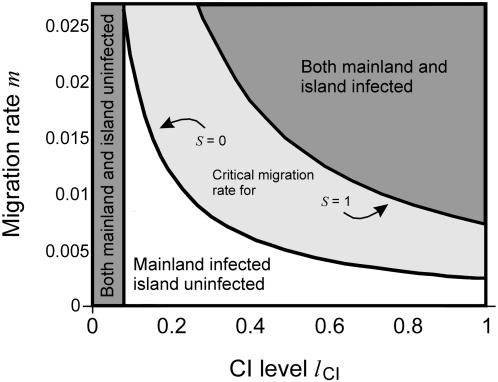
Critical migration rates. Shown are the critical migration rates (highest migration rates below which post-zygotic isolation stably persists) as a function of the CI level for *f* = 0.1 and *s* = 0 or *s* = 1. The parameter space consists of three regions. If *l*_CI_<*f* then *Wolbachia* is lost in both populations. If *l*_CI_≥*f* then *Wolbachia* persists on the mainland and can spread on the island only if migration is above the critical migration rate but cannot if migration is below this threshold. The critical migration rate separates the latter region from the other two and increases with increasing selection coefficient *s*.

Flor et al. [37] analyzed the stability of the infection polymorphism and unidirectional CI in the absence of local adaptation. It was demonstrated analytically that the parameter space spanned by the CI level and the migration rate consists of three qualitatively different regions: (a) a region where *Wolbachia* goes to extinction in both populations, (b) a region where *Wolbachia* spreads to fixation in both populations, and (c) a region where an initially uninfected island remains at low infection frequencies despite migration from an infected mainland (fig. 2). The critical migration rate separates the third region from the other two. For low CI levels of *l*_CI_ *<f*, critical migration rates are zero because CI is not sufficiently strong to offset the fecundity reduction. However, if CI levels are larger than the fecundity reduction, positive critical migration rates are observed. An interesting finding from that study is that high critical migration rates are reached for rather low levels of CI and is the highest for *l*_CI_ *= f*.

In the present study we investigate how local adaptation affects the stability of infection polymorphism and unidirectional CI. Our results demonstrate that generally the critical migration rate increases with increasing selection coefficient *s*. This is because local adaptation favors residents in comparison to migrants, and the infection type and selected locus tend to be coupled in association disequilibrium, which imparts a selective advantage to both in the resident population (uninfected cytotypes are associated with the selectively favored allele *T*_2_ in the island population). Local adaptation can result in comparatively high critical migration rates (fig. 2). If, for example, the CI level is *l*_CI_ *= *0.5 and *f = *0.1 then the critical migration rate for *s = *0 is *m_c_ = *0.5%, but for *s = *1 it is *m_c_ = *1.6%. Note that *s* = 1 corresponds to a two-fold fitness advantage for the resident allele. This shows that local adaptation significantly stabilizes postzygotic isolation induced by unidirectional CI.

#### Analytical Analysis

Telschow et al. [26] determined analytically critical migration rates in a two population model with bidirectional CI using standard fixpoint analysis. This method can be applied to unidirectional CI if no viability selection at the trait locus is considered (for details see [37]). For the mainland-island scenario considered in the present study the critical migration rate is
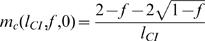
(1)if *l*_CI_≥*f*. If *l*_CI_ *<f* then the *Wolbachia* infection cannot persist stably on the mainland and the critical migration rate is zero (see also fig. 2).

Formula (1) does not incorporate the effects of local adaptation. In this case, it is not possible to determine the critical migration rate analytically. However, we were able to derive analytically a lower estimation of *m_c_* (see Text S2 for a proof). For *l*_CI_≥*f* and *s*≥0 it holds that
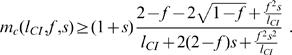
(2)Note that the right-hand side of equation (2) reduces for *s* = 0 to formula (1).

Three conclusions can be drawn from inequality (2). First, adding a locus under local selection results generally in an increase of the critical migration rate. A straightforward calculation shows that *m_c_*(*l*_CI_, *f*, *s*)*>m_c_*(*l*_CI_, *f*, 0) for all *s>*0. Second, linearization of *m_c_* around *s = *0 reveals that *m_c_* (*l_CI_, f, s*)≈(1+*s*)*m_c_* (*l_CI_, f,* 0) if *s* is small. Third, the critical migration rate converges to one if *s* goes to infinity. The important implication here is that large *s* result in large critical migration rates.

### Gene Flow Reduction and Local Adaptation

Next we investigate how the infection polymorphism and unidirectional CI affect the genetic divergence at the locus under differential selection and interpret the results in terms of the concept of the effective migration rate [27], [41], [42].

#### Effective Migration Rate

We follow Telschow et al. [42] and use a definition of the effective migration rate that was introduced to measure gene flow on weakly selected loci. Considering a locus under local adaptation, the effective migration rate between two populations, where one is infected with *Wolbachia*, is defined as the migration rate that-in a scenario without *Wolbachia*-would result in the same equilibrium frequencies at that locus. Note that the scenario where both populations are infected yields the same divergence at the selected locus as the scenario without *Wolbachia*, and the effective migration rate is equal to the real migration rate.

Telschow et al. [27] derived for bidirectional CI an analytical approximation for the effective migration rate. Here, we point out that this approach is applicable to the case of unidirectional CI (see supplementary material (Text S3) for details). Under the assumption that migration rate *m* and selection coefficient *s* are small the effective migration rate between an infected mainland and an uninfected island is in good approximation
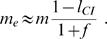
(3)The key point for deriving this formula is to consider the matriline of female migrants over successive generations. Formula (3) shows that a CI level of 0.5 results in a reduction of the effective migration rate of more than 50%, a CI level of 1 results in zero gene flow. This is counterintuitive because only infected male migrants suffer from CI. However, the recursive nature of reduction in effective migration rate shows that gene flow is inhibited in the matriline too because each generation sons of female migrants inherit the infection and therefore suffer from CI, thus further reducing input of genes from migrant descendants into the population.

#### Local Adaptation

**Figure 3:**
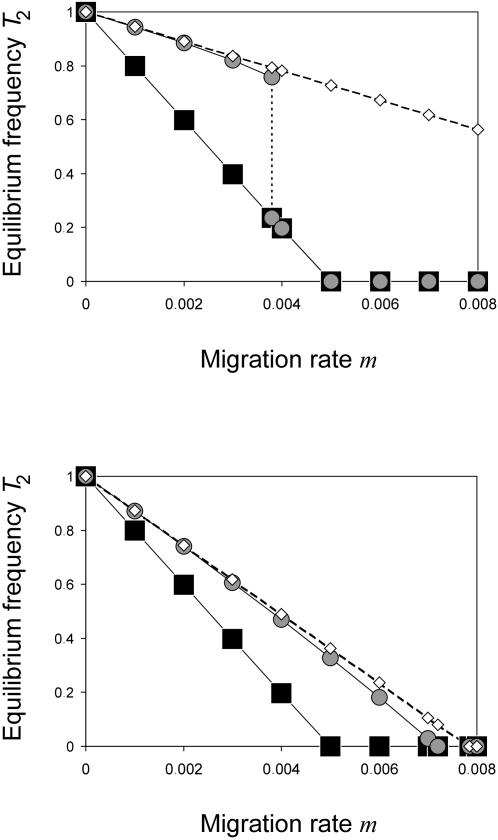
Equilibrium frequencies of *T*_2_ on the island. Black symbols indicate that both mainland and island are infected with *Wolbachia*. The frequencies were calculated analytically with formula (5). Gray symbols show numerically determined equilibrium frequencies for starting conditions with an infected mainland and an uninfected island. White diamonds show approximations of the latter case using formula (8). Parameters are *f* = 0.1, *s* = 0.005 in both graphs, *l*_CI_ = 0.7 in *(a)*, and *l*_CI_ = 0.3 in *(b)*.

We calculated the frequencies of the locally adapted *T*_2_ allele on the island for the scenario where the mainland is infected but the island not, and compared them with the scenario where both mainland and island are infected. Figure 3 shows that the former scenario results in higher genetic divergence between the populations at the selected locus over a broad range of migration rates. This holds true for varying selection coefficients (results not shown). The basic reason is that the selected locus and the cytotype are in linkage disequilibrium. On the island, the positively selected allele *T*_2_ is associated with uninfected individuals. Because uninfected individuals suffer less from CI, this linkage disequilibrium boosts the frequency of the *T*_2_ allele. However, these linkage disequilibria occur only as long as *Wolbachia* cannot spread on the island. If migration is above the critical migration rate, *Wolbachia* spreads on the island and no differences in *T*_2_ allele frequencies are seen (fig. 3a). Figure 3b shows that there is a range where local adaptation disappears entirely on the island in the absence of CI, but is maintained in the presence of CI. For example, if *m* = 0.005 and *s* = 0.005 then the *T*_2_ allele goes to extinction when the island is infected but reaches high frequencies of 37.7% when the island is uninfected.

#### Analytical Analysis

The dynamics of the *T*_2_ allele can be investigated analytically using the effective migration rate introduced above. First, let us consider the simplest situation where both mainland and island are uninfected. Note that the resulting dynamics do not differ from the situation where both populations are infected. Let 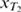 and 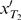 denote the frequencies of the *T*_2_ allele on the island in subsequent generations. Then it holds that
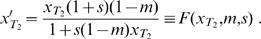
(4)To determine the equilibrium frequency 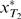 of the system, we solve equation (4) for 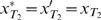. This yields
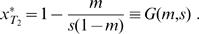
(5)Next, we consider the general case which includes the situation with an infected mainland and an uninfected island. The dynamics are now more complex and cannot be solved analytically anymore. However, good analytical approximations can be achieved using the effective migration rate. Our simple approach is to substitute *m* by *m_e_* in equations (4) and (5). This yields the following approximation of the dynamics of *T*_2_ and its equilibrium frequencies,
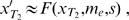
(6)

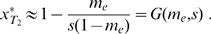
(7)Combining (3) and (7) under the assumption that *m* is small results in the following approximation for the equilibrium frequencies of *T*_2_

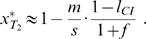
(8)As illustrated in figure 3, formula (8) yields good approximations of the real equilibrium frequencies of *T*_2_ calculated by computer simulations. Note that formula (8) applies only to the situation where migration is below the critical migration rate (see fig. 3a). If migration is above this critical value, the equilibrium frequencies are given by (5).

A comparison of formula (5) and (8) demonstrates that *T*_2_ always reaches higher frequencies in the scenario with an uninfected island than in the scenario with an infected island (see also fig. 3). Taking the difference of both we see that the increase in frequency of the *T*_2_ allele is linear in *l*_CI_ and computes for small *m* to 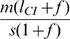. Moreover, the results demonstrate that unidirectional CI significantly enlarges the range of parameters where local adaptation on the island is possible. In the scenario with both mainland and island infected the *T*_2_ allele gets lost on the island if *m*≥*s*/(1+*s*), whereas it can persist on an uninfected island as long as *m_e_*≈*m*(1−*l*_CI_)/(1+*f*)<*s*/(1+*s*) .

### Premating Isolation

In the third part of the results section we investigate under which circumstances postzygotic isolation (caused by unidirectional CI) selects for premating isolation. Generally, the migration rate is chosen to be below the critical migration rate.

#### Equilibrium Frequencies

**Figure 4:**
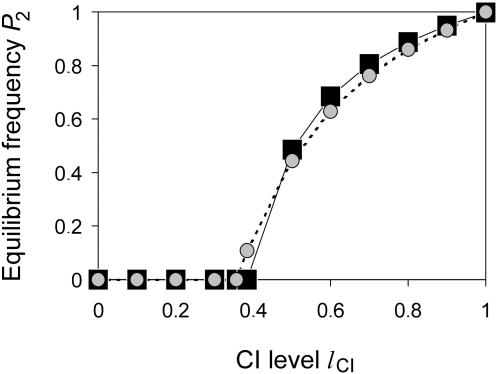
Equilibrium frequencies of *P*_2_ after introduction on the island with the low frequency of 0.1%. Black squares indicate simulation results, gray circles values determined by formula (16). The figure illustrates the finding that *P*_2_ spreads on the island if the CI level is above a threshold but goes to extinction if the CI level is below. Parameters are *a*_2_ = 10, *f* = 0.1, *s* = 0.1, *m* = 0.003.

To investigate the impact of unidirectional CI on genetic divergence we first determined equilibrium frequencies of the system after introduction of the preference allele *P*_2_. In all simulations performed, a minimal level of CI was necessary for *P*_2_ to spread. If the mating preference strength is *a*_2_ = 10, as shown in figure 4, then *P*_2_ goes to extinction if the CI level is below 0.384 but spreads on the island if CI is above this threshold. The frequencies of *P*_2_ increase with increasing CI level and reach high frequencies close to one for high levels of CI.

The minimal CI level for *P*_2_ to spread has its roots in two opposing forces. CI creates a selective advantage for *P*_2_ but in order to spread this advantage must be sufficient to offset the gene flow from the mainland. Note that choosiness itself creates a double advantage for *P*_2_ females. First, mating with the locally better adapted *T*_2_ males results in locally better adapted offspring. Second, matings with *T*_2_ males result less often in CI because *T*_2_ males are less likely to be infected with *Wolbachia*. The latter can be a huge advantage because *P*_2_ females are mostly not infected with *Wolbachia*. From the female's perspective, the advantage of being choosy is therefore not only to find a male that fits the environment but also to find a male that is compatible with one's own cytotype. Because the selected allele and cytotype are in association, female choice of the selected allele enhances chances of mating with a compatible cytotype.

#### Thresholds for Divergence at the Preference Locus

**Figure 5:**
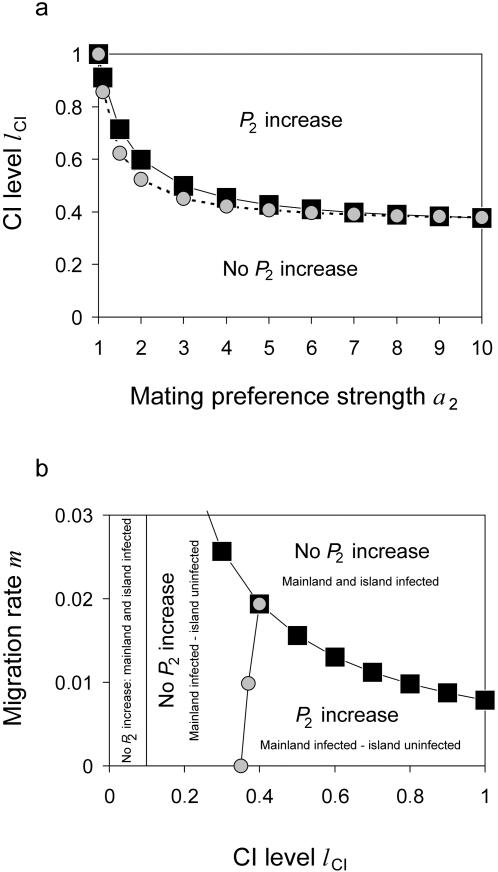
Thresholds for the spread of the mating preference mutant. *Graph (a):* The parameter plane spanned by the preference strength and the CI level consists of two areas. For low levels of CI, *P*_2_ cannot spread no matter how strong the mating preference is. For CI levels above a certain threshold, however, *P*_2_ spreads on the island. Black squares indicate simulation results, gray circles values determined by formula (17). *Graph (b):* The parameter space spanned by the CI level and the migration rate consists of four areas. First, if *l*_CI_<*f* then mainland and island are uninfected and *P*_2_ cannot spread. Second, if *m*>*m_c_* then both mainland and island are infected and *P*_2_ cannot spread. Third and fourth, if *l*_CI_>*f* and *m*<*m_c_* then *P*_2_ can spread if the CI level is above a threshold but cannot if the CI level is below. Parameters are *f* = 0.1 in both graphs, *m* = 0.001, *s* = 0.1 in *(a)*, and *a*_2_ = 10, *s* = 1 in *(b)*.

In what follows we screened the parameter space more generally. Figure 5a shows the plane spanned by the preference strength and the CI level. The mutant at the preference locus *P*_2_ cannot spread for low CI levels no matter how strong the mating preference is. However, if the CI level is above a threshold, *P*_2_ spreads on the island. The threshold CI level decreases with increasing preference strength *a*_2_. Both increasing viability selection and decreasing migration reduce this threshold further (results not shown). Qualitatively similar results were presented by Servedio [13] for a mainland-island model of reinforcement where postzygotic isolation is caused by nuclear epistatic interactions.

Figure 5b shows the plane spanned by the CI level and the migration rate. Four areas can be seen. First, if *l*_CI_<*f* then both mainland and island are uninfected and *P*_2_ cannot spread. Second, if *m*>*m_c_* then both mainland and island are infected and *P*_2_ cannot spread. Third and fourth, if *l*_CI_>*f* and *m*<*m_c_* then *P*_2_ can spread if the CI level is above a threshold but cannot if the CI level is below. The important implication of figure 5b is that moderate CI levels select for premating isolation in a larger part of the parameter space than high CI levels. This is because infection differences between the mainland and island population are maintained for higher *m* when the CI level is not too high.

#### Analytical Analysis

As discussed above, there are two opposing forces acting on the preference mutant *P*_2_. On the one side, *P*_2_ individuals have a selective advantage over *P*_1_ individuals because they are less often involved in incompatibility matings. On the other side there is permanent gene flow of the *P*_1_ allele from the mainland. Here, we formalize the verbal reasoning and derive heuristic formulae for the calculation of *P*_2_ allele frequencies.

We use the above defined function *F* = *F*(*x*, *m*, *s*) to describe selection and migration acting on the preference locus. In order to apply the function *F* to the *P*_2_ dynamics we will define an “effective selection coefficient” *s_e_* that describes the selective advantage of *P*_2_ over *P*_1_. In addition, we take into account that gene flow is appropriately described by the effective migration rate *m_e_*. As we will show, the following equations are good approximations for the dynamics and equilibrium frequencies of the *P*_2_ allele,
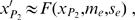
(9)

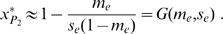
(10)Here, 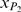 and 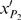 denote the frequencies of the *P*_2_ allele on the island in subsequent generations, and 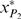 its equilibrium frequency.

In order to define an effective selection coefficient for a rare *P*_2_ allele in a *P*_1_ population we determine the average fitness of the respective alleles. First, we consider the case that *P*_2 _females mate exclusively with *T*_2_ males, i.e. *a*_2_ = ∞. Under this assumption, there is strong linkage disequilibrium between *P*_2_ and *T*_2_. Let *x_W_* denote the frequency of *Wolbachia* on the island. Then the average fitness of *P*_1_ is 1−*l*_CI_ *x_W_*. The fitness of *P*_2_ individuals is 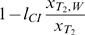, where 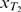 denotes the frequency of *T*_2_ on the island and 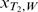 the frequency of infected *T*_2_ individuals. As the effective selection coefficient we take the difference of both,
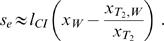
(11)In order to get a useful approximation of *s_e_*, we assume that 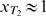. This is justified for small *m*. Denoting the frequency of infected *T*_1_ individuals with 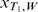 we get
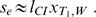
(12)As shown in the supplementary material (Text S4), a good approximation of 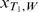 for small *s* is 2*m*/(1+*f*). Substitution into formula (12) yields the following useful approximation for the effective selection coefficient which depends only on the parameters of the system,
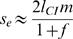
(13)In a next step, we consider the case where *a*_2_<∞. In general, the selective advantage of *P*_2_ over *P*_1_ is reduced by smaller *a*_2_. This is because *P*_2_ females are more likely to be involved in incompatibility matings if *a*_2_ is low, whereas the fitness of *P*_1_ individuals stays the same. For finite *a*_2_ the fitness of a rare *P*_2_ allele is 
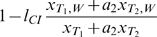
. This expression simplifies to 
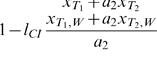
 if we assume, as above, that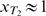. Again, we set as the effective selection coefficient *s_e_* the fitness difference of the two mating preference alleles. This results in
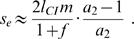
(14)Note that if *a*_2_ goes to infinity, formula (14) correctly reduces to formula (13). To achieve approximations for *P*_2_ equilibrium frequencies, we substitute (3) and (14) in (10). This yields
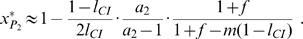
(15)For small *m* formula (15) reduces to
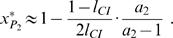
(16)A consequence of formula (16) is that *P*_2_ can spread into the population only if
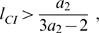
(17)but goes to extinction if the CI level is below this threshold value. Note that the threshold CI level decreases with increasing preference strength and converges to 1/3 if *a*_2_ goes to infinity. Figure 4 and 5a illustrate formulae (16) and (17) and demonstrate that they approximate the numerically determined values well. The approximations are especially good when mating preference is strong.

## Discussion

In this article we investigated the role of *Wolbachia*-induced unidirectional CI on genetic divergence and reinforcement using a mainland-island model. We demonstrated analytically that (a) the infection polymorphism between populations is stabilized by local adaptation, (b) gene flow between the populations is reduced but divergence at a selected locus enhanced, and (c) unidirectional CI selects for premating isolation in a mostly uninfected island population receiving migration from a *Wolbachia*-infected mainland. Interestingly, premating isolation is most likely to evolve if levels of incompatibility are intermediate, if the infection causes fecundity reductions or *Wolbachia* transmission is incomplete. This is because under these circumstances an infection pattern with an infected mainland and a mostly uninfected island can persist in the face of comparably high migration. We anticipate that if additional locally adapted alleles accumulate in the island population then stability will persist at even higher levels of CI.

These findings generally support the view that *Wolbachia*-induced unidirectional CI could be a factor in host divergence and speciation, but only under certain conditions. The role of *Wolbachia* would be to reduce gene flow between populations, allowing genetic divergence for locally adaptive traits, and to select for premating isolation. Furthermore, the association of infection status with locally adapted alleles and local male traits creates positive selection for females with local mate preference, both because of the advantages of local adapted genotypes and of mating with compatible cytotype males.

Our results suggest that peripheral populations that have lost their *Wolbachia* infection are able to maintain local adaptation in the face of migration better than peripheral populations that maintain their infection (fig. 1b). The threshold migration rate for maintenance of the infection difference is therefore increased, and premating isolation more readily evolves. This means that if recurrent peripheral populations occur, it is the ones that lose their *Wolbachia* that are more likely to diverge into new species. This could be considered a form of population selection, where populations that lose their infections are better able to “resist” gene flow and therefore locally adapt and evolve into new species. The scenario is not simply hypothetical. North American populations of the fire ant *Solenopsis invicta*, which were established by presumably small founding populations due to human transport, are devoid of *Wolbachia*, whereas South American source populations show infection polymorphisms [43], [44]. Similarly, Atlantic coast populations of the beetle *Chelymorpha alternans* are infected with two *Wolbachia* strains, whereas some Pacific coast populations have lost one of the *Wolbachia* [22]. Thus, the scenarios envisioned here could occur in nature. A counter example is the spread of *Wolbachia* in uninfected populations of *Drosophila simulans* in uninfected North American populations [33].

Our results also have important ramifications for classical reinforcement theory. A standard critique in reinforcement is that gene flow might overwhelm the selective advantage of being choosy [45]. Although previous work has shown that reinforcement is possible (e.g. [12], [13], [46]), most of the studies are purely simulation based (but see [47]). In the present study we developed a new method to analyze the reinforcement dynamics analytically. In this approach gene flow is modeled by an effective migration rate and the advantage of being choosy by an effective selection coefficient. We believe that this approach is also applicable to other reinforcement scenarios, i.e. with postzygotic isolation caused by nuclear mating incompatibilities.

The possible role of *Wolbachia* in host speciation has generated some controversy over the past decade [3], [6], [18], [29], [48]. One major criticism against *Wolbachia*-induced speciation is that in most systems *Wolbachia* cannot totally prevent gene flow because both levels of cytoplasmic incompatibility and transmission rates are incomplete [49]. Our results show that unidirectional CI is most likely to select for premating isolation if levels of incompatibility are intermediate, if the infection causes fecundity reductions or *Wolbachia* transmission is incomplete (see Text S5). The fact that in most systems cytoplasmic incompatibility is incomplete broadens therefore the conditions under which unidirectional CI selects for premating isolation and is, based on our analysis, rather an argument for a role of *Wolbachia* in host speciation than against it.

A second criticism is that unidirectional CI is unlikely to be stable. Crucial in our speciation scenario is that infected and uninfected host populations can stably persist in the face of migration. In a previous study we have demonstrated that both fecundity reduction and incomplete transmission rate can prevent *Wolbachia* from spreading [37]. The observed critical migration rates, however, are relatively low, thus narrowing the range where unidirectional CI as a single factor could promote host speciation. We expect that unidirectional CI is most likely to promote speciation if it acts in concert with other genetic factors that stabilize infection differences and enhance critical migration rates. In the present study we have shown that locally adapted alleles substantially stabilize postzygotic isolation (fig. 2). Other factors that will likely stabilize infection differences and postzygotic isolation are cytoplasmic sex ratio distorters [50], nuclear based mating incompatibilities (Telschow, unpublished results), and the accumulation of multiple adaptive genes within the diverging populations. The latter would be particularly important when epistatic interactions among adapted loci occur. As shown for the single adapted locus case and for pairwise genetic incompatibilities [27], [28, here], these effects further enhance the association of infection type with adapted loci, increasing the frequencies of each in the presence of immigration.

A third criticism of the possible role of unidirectional CI in divergence and speciation is that nuclear gene flow is reduced or prevented only in one direction (from the infected to the uninfected population), whereas nuclear genes flow freely from the uninfected to the infected population. We have not analyzed this effect in this article, since we considered only one-way migration from the mainland to the island population. Therefore, the model presented here is likely to apply only to conditions where migration from a small island population to a large mainland population is much lower than in the other direction. However, we also investigated an extended model with two populations and migration in both directions (see [51] for details). For this extended model the effective migration rate from an uninfected population to an infected population can be approximated by *m*/(1+*l*_CI_) (see formula (1) in [27]); a CI level of *l*_CI_ = 1, for example, results in 50% gene flow reduction. As shown in the results, the effective migration rate from an infected to an uninfected population is approximately *m*(1−*l*_CI_) and therefore generally results in higher gene flow reduction. If the CI level is one then gene flow is prevented totally. These results together show that gene flow between an infected and an uninfected population is reduced in both directions but that this reduction is asymmetric. The resulting asymmetric gene flow reduction between the populations, however, affects the pattern of adaptation in the *Wolbachia* host; adaptation is favored in the uninfected population but impeded in the infected population. A comparison of the mainland-island model with the two-way migration model reveals that back migration facilitates local adaptation and the spread of mating preference mutants on the island. Under certain parameter values, asymmetric gene flow and runaway sexual selection leads to an increase of the introduced female preference allele and fixation of the preference mutant or the preferred male trait in both populations, whereas under other parameter values it leads to divergence in the two populations in preference and male trait alleles. This is in concordance with previous theoretical studies [6], [13]).

The full impact of unidirectional CI on host speciation in nature will likely involve more complex population structures than modeled here. The results on the critical migration rates suggest that *Wolbachia* infections might occur in mosaic patterns with infected and uninfected patches close by. We have shown that under these circumstances unidirectional CI selects for female mating preferences in uninfected patches facing migration from infected patches. This observation lets us state the general prediction that species infected with *Wolbachia* (causing unidirectional CI) will evolve premating discrimination more rapidly than their uninfected sibling species. So far there is one case in concordance with this view. The mushroom feeding *Drosophila recens* is infected with unidirectional CI inducing *Wolbachia* and shows, when allopatric populations are considered, significantly stronger mating discrimination than the uninfected sibling species *D. subquinaria* [5], [36]. Of course, this case alone is not sufficient to test our hypothesis. Therefore we suggest a comparative study similar to the work of Coyne and Orr [52], [53]. These authors screened over 171 pairs of *Drosophila* species and concluded that sym-or parapatric pairs show larger mate discrimination than allopatric pairs. A re-analysis of these data with infection status added may reveal whether earlier evolution of mate discrimination is associated with closely related species pairs where one is infected with *Wolbachia*.

Our model scenario involves a full allopatric phase during which the locally adapted allele spreads on the island. The mutant at the preference locus is introduced after secondary contact is established. An alternative model setting would allow a certain amount of migration from the start. It is important to remark that the full allopatry at the beginning is not a necessary condition for reproductive isolation to evolve. A modification of our model with migration from the start does not change the results qualitatively as long as migration is below the critical migration rate.

In this study we analyzed the question whether a single *Wolbachia* strain causing unidirectional CI can select for local adaptation and premating isolation. Previously, it was shown that two *Wolbachia* strains causing bidirectional CI promote local adaptation and select for rapid premating isolation under a broad variety of conditions [6]. This is mainly because bidirectional CI can persist up to high critical migration rates of over 15% per generation [26]. As shown in this study, critical migration rates for unidirectional CI are much lower, resulting in a comparatively weak selection for premating isolation. These results suggest that once bidirectional CI is established in a system, its impact on host speciation is much stronger than that of unidirectional CI. Nevertheless, the full significance of unidirectional CI for host speciation might be bigger than that of bidirectional CI. First of all, unidirectional CI is much more common in nature because it requires that only one population is infected whereas bidirectional CI requires different infections in both populations. Furthermore, in case of bidirectional CI, one *Wolbachia* strain is inevitably eliminated if postzygotic isolation is destroyed. Postzygotic isolation can be reestablished only if the system becomes infected again with a second *Wolbachia* strain, a rather unlikely event. In the unidirectional CI scenario, however, reestablishment of postzygotic isolation involves only the loss of the infection in an island population, an event which is much more likely to reoccur. In fact recurrent establishment of peripheral isolate populations from a central source population could produce the circumstances for repeated opportunities for local adaptation and speciation. Our results suggest that when these conditions occur, those peripheral populations that have lost their infections are more likely to maintain locally adapted alleles and to evolve reinforcement of mate discrimination.

In summary, our results demonstrate that a stable coexistence between infected and uninfected host populations is possible if migration is below a critical migration rate. Under these circumstances unidirectional CI acts as a postzygotic isolation mechanism and selects for local adaptation and premating isolation under a variety of conditions. These results generally suggest that unidirectional CI could be a factor in speciation processes of arthropods.

## Supporting Information

Text S1 Model description.
(0.05 MB DOC)

Text S2 Lower estimation of the critical migration rate.
(0.04 MB DOC)

Text S3 Formula for the effective migration rate.
(0.02 MB DOC)

Text S4 Equilibrium Frequencies of infected *T*_1_ Individuals
(0.03 MB DOC)

Text S5 Incomplete transmission.
(0.03 MB DOC)
