# Supplementary material for: Wolbachia-Induced Unidirectional Cytoplasmic Incompatibility and Speciation: Mainland-Island Model
Source: PLoS One. 2007 Aug 8;2(8):e701. doi: 10.1371/journal.pone.0000701 (PMC1934337; doi:10.1371/journal.pone.0000701)
Supplement: Text S1 — Model description. (0.05 MB DOC) [file pone.0000701.s001.doc]

**S1 Model Description**

We designed a haploid sexual model to describe the interactions between mating preference and *Wolbachia*-induced unidirectional CI. Two nuclear host loci are considered, one locus for female mating preference with the two alleles *P*_1_ and *P*_2_, and one male trait locus with the alleles *T*_1_ and *T*_2_. We consider an island population receiving migration from a mainland population. Organisms have a life cycle consisting (in that order) of migration, viability selection, sexual selection, and reproduction. The first three steps are assumed to happen in the haploid phase of the organisms. The reproduction step includes the formation of a diploid zygote and the production of female and male gametes with a primary sex ratio of 1:1. There is no overlap between generations.

Individuals are described by both nuclear genotype and cytotype. Since there are two possible cytotypes (infected with *Wolbachia* and uninfected) and four different nuclear genotypes (*P*_1_*T*_1_, *P*_1_*T*_2_, *P*_2_*T*_1_, *P*_2_*T*_2_) we have eight different nucleocytotypes. The intergenerational change of these frequencies is described by a system of eight coupled difference equations. We denote by *x_i,j,k_* the frequencies of nucleocytotype (*i*, *j*, *k*) in the island population and by *y_i,j,k_* in the mainland population, where *i* = 1 means infected with *Wolbachia*, *i* = 2 means uninfected, and (*j, k*) indicates nucleotype *P_j_T_k_*. To get $x'_{i,j,k}$ and $y'_{i,j,k}$, the frequencies of the nucleocytotypes in the next generation, we take into account the effects of migration, viability selection, sexual selection, and cytoplasmic incompatibility. Thereby, we assume a starting condition where all mainland organisms have the nucleotype *P*_1_*T*_1_ and are infected with *Wolbachia*. In order to state the equations we first define the following weighting factors:

(18) $F_{i}=\left\{\begin{matrix} 1-f \\ 1 \end{matrix}\right.\begin{matrix} \\ \end{matrix}\begin{matrix} for \\ for \end{matrix}\begin{matrix} \\ \end{matrix}\begin{matrix} i=1 \\ i=2 \end{matrix}$,

(19) $V_{i,j}=\left\{\begin{matrix} 1 \\ 0 \end{matrix}\right.\begin{matrix} \\ \end{matrix}\begin{matrix} \\ \end{matrix}\begin{matrix} \\ \end{matrix}\begin{matrix} for \\ for \end{matrix}\begin{matrix} \\ \end{matrix}\begin{matrix} i=j \\ i\neq j \end{matrix}$,

(20) $L_{i,j}=\left\{\begin{matrix} 1-l_{CI} \\ 1 \end{matrix}\right.\begin{matrix} \\ \end{matrix}\begin{matrix} for \\ for \end{matrix}\begin{matrix} \\ \end{matrix}\begin{matrix} i=1,j=2 \\ else \end{matrix}$,

(21) $I_{i,j,k}=\left\{\begin{matrix} 0 \\ 0.5 \\ 1 \end{matrix}\right.\begin{matrix} \\ \\ \end{matrix}\begin{matrix} \\ \\ \end{matrix}\begin{matrix} for \\ for \\ for \end{matrix}\begin{matrix} \\ \\ \end{matrix}\begin{matrix} i=j\neq k \\ i\neq j \\ i=j=k \end{matrix}$.

The weighting factors describe the fecundity costs in infected females (*F_i_*), the maternal transmission of cytoplasmic *Wolbachia* (*V_i,j_*), *Wolbachia*-induced cytoplasmic incompatibility (*L_i,j_*), and the inheritance of nuclear genes (*I_i,j,k_*). The parameter *l*_CI_ is called CI level and defined as the proportion of zygotes that die if an uninfected egg is fertilized by sperm from an infected father. We are now able to state the recursion formula. The intergenerational transition is split into four steps, migration, viability selection, sexual selection, and reproduction.

*Migration:* The first step in the life cycle of the hosts is the migration of the haploid individuals. Thereby a fraction of the island population is replaced by individuals from the mainland. We denote this fraction of migrants in the island population by *m*. It holds that

(22) $x_{i,j,k}^{+}=(1-m)x_{i,j,k}+my_{i,j,k}$

*Viability selection:* The second step in the life cycle is the selection at the male trait locus *T*. On the mainland, *T*_1_ is favored and therefore fixed. On the island, however, individuals with genotype *T*_2_ have a (1+*s*)-times higher fitness than individuals with genotype *T*_1_. Selection at the *T* locus is described by equation (23). Thereby *W*_1_ denotes the sum of all nominators of (23).

(23) $x_{i,j,k}^{++}=\left\{\begin{matrix} \frac{x_{i,j,k}^{+}}{W_{1}} \\ \frac{(1+s)x_{i,j,k}^{+}}{W_{1}} \end{matrix}\right.\begin{matrix} \\ \end{matrix}\begin{matrix} for \\ for \end{matrix}\begin{matrix} \\ \end{matrix}\begin{matrix} k=1 \\ k=2 \end{matrix}$.

*Weighting factors of sexual selection:* To model mating preference we follow Servedio [13] and introduce the weighting factors *X_i,j_*. These are a measure of how often females of genotype *P_i_* mate with males of genotype *T_j_*. The parameter *a*_2_ indicates the mating preference strength of *P*_2_. Females of genotype *P*_1_ show no mating preference (*a*_1_ = 1). Let $x_{T_{2}}^{++}$ be the frequency of allele *T*_2_ after viability selection, then the weighting factors can be defined as

(24) $X_{i,j}=\left\{\begin{matrix} \frac{a_{2}}{1+(a_{2}-1)x_{T_{2}}^{++}} \\ 1 \\ \frac{1}{1+(a_{2}-1)x_{T_{2}}^{++}} \end{matrix}\right.\begin{matrix} \\ \\ \end{matrix}\begin{matrix} \\ \\ \end{matrix}\begin{matrix} for \\ for \\ for \end{matrix}\begin{matrix} \\ \\ \end{matrix}\begin{matrix} i=j=2 \\ i=1 \\ i=2,j=1 \end{matrix}$.

*Reproduction:* The last step in the life cycle is the reproduction of new haploid offspring. This includes sexual selection, fecundity costs of infection, *Wolbachia* transmission, cytoplasmic incompatibility, and the inheritance of nuclear genes. To get the frequencies of certain nucleocytotypes in the next generation we first sum over all possible matings and weigh the outcome subsequently with the factors *X_i,j_*, *F_i_*, *V_i,j_*, *L_i,j_*, and *I_i,j,k_*. The frequencies in the next generation are achieved by dividing the weighed outcomes by the average fitness *W*_2_.

(25) $x'_{\alpha,\beta_{1},\beta_{2}}=\frac{1}{W_{2}}\sum_{i,j} \sum_{r_{1},r_{2},k_{1},k_{2}} x_{i,r_{1},r_{2}}^{++}x_{j,k_{1},k_{2}}^{++}X_{r_{1},k_{2}}F_{i}V_{i,\alpha}L_{j,\alpha}I_{r_{1},k_{1},\beta_{1}}I_{r_{2},k_{2},\beta_{2}}$
