# Supplementary material for: Wolbachia-Induced Unidirectional Cytoplasmic Incompatibility and Speciation: Mainland-Island Model
Source: PLoS One. 2007 Aug 8;2(8):e701. doi: 10.1371/journal.pone.0000701 (PMC1934337; doi:10.1371/journal.pone.0000701)
Supplement: Text S2 — Lower estimation of the critical migration rate. (0.04 MB DOC) [file pone.0000701.s002.doc]

**S2 Lower Estimation of the Critical Migration Rate**

In order to derive an estimation of the critical migration rate, we use a simplified system and then follow the standard fixpoint analysis according to [26]. Let all migrants carry trait *T*_1_ and be infected with *Wolbachia*, and let all residents harbor trait *T*_2_. Furthermore, let all offspring inherit *T*_2_. Note that because the latter assumption is beneficial to the migrants, a spread of the infection on the island is facilitated. Denoting the *Wolbachia* frequency in consecutive generations by *x_W_* and $x'_{W}$, the dynamics of this simplified system can be written as

(26) $x_{W}^{+}=\frac{(1-m)(1+s)x_{W}+m}{1+(1-m)s}$,

(27) $x'_{W}=\frac{(1-f)x_{W}^{+}}{1-fx_{W}^{+}-l_{CI}x_{W}^{+}(1-x_{W}^{+})}$,

where the former equation describes the migration and viability selection steps, and the latter equation the reproduction step. Requiring $x_{W}=x'_{W}$, the fixpoints of the system can be calculated:

(28) $x_{1}^{*}=1$,

(29) $x_{2,3}^{*}=\frac{f+R-l_{CI}m\pm\sqrt{f^{2}R^{2}+2fl_{CI}mR-l_{CI}m(4R-l_{CI}m)}}{2l_{CI}(1-m)(1+s)}$,

where *R* = *R*(*m*, *s*) = 1+(1-*m*)*s*. The critical migration rate is the migration rate where *x*_2_^*^ = *x*_3_^*^ and computes to $(1+s)\frac{2-f-2\sqrt{1-f}+\frac{f^{2}s}{l_{CI}}}{l_{CI}+2(2-f)s+\frac{f^{2}s^{2}}{l_{CI}}}$. If migration is above this critical rate, *x*_1_^*^ is the only stable fixpoint and *Wolbachia* will inevitably spread to fixation. However, if migration is below the critical rate, $x_{2,3}^{*}$ are real numbered, and the island may maintain a low infection frequency status (i.e. stay at $x_{3}^{*}$) despite migration from the infected mainland.

As argued above, the spread of *Wolbachia* on the island is facilitated in the simplified system as compared to the full system. Therefore, it holds that for the full system the critical migration rate can be estimated by

(30) $m_{c}(l_{CI},f,s)\geq(1+s)\frac{2-f-2\sqrt{1-f}+\frac{f^{2}s}{l_{CI}}}{l_{CI}+2(2-f)s+\frac{f^{2}s^{2}}{l_{CI}}}$

if *l*_CI_ > *f* > 0 and *s* ≥ 0.
