# Supplementary material for: Wolbachia-Induced Unidirectional Cytoplasmic Incompatibility and Speciation: Mainland-Island Model
Source: PLoS One. 2007 Aug 8;2(8):e701. doi: 10.1371/journal.pone.0000701 (PMC1934337; doi:10.1371/journal.pone.0000701)
Supplement: Text S3 — Formula for the effective migration rate. (0.02 MB DOC) [file pone.0000701.s003.doc]

**S3 Formula for the Effective Migration Rate**

We follow Telschow et al. [27] to derive an approximation for the effective migration rate from a *Wolbachia*-infected mainland to a (mainly) uninfected island population. We assume that each migrant mates with an (uninfected) resident. Therefore all migrants are involved in incompatibility matings. This assumption is justified for low migration rates. Further, we neglect the effects of local adaptation acting at the *T* locus.

Because all migrants harbor *Wolbachia*, male migrants have, on average, *l*_CI_ fewer offspring in an incompatibility mating, whereas migrant females have *f* fewer offspring. In summary, this leads to an effective reduction of the migration after the first generation by (*f* +*l*_CI_)/2. In the second generation, incompatibility matings occur only in the matriline, resulting in a reduction by (*f*+*l*_CI_)(1-*f*)/4. Our full approximation of the effective migration rate *m_e_* includes gene flow reduction in all subsequent generations and computes to

(31) $\frac{m_{e}}{m}\approx1-\frac{1}{2}(f+l_{CI})\sum_{n=0}^{\infty} \left[\frac{1-f}{2}\right]^{n}$

By using the formula for the geometric series, we get the approximation of the effective migration rate used in the results,

(32) $\frac{m_{e}}{m}\approx1-\frac{f+l_{CI}}{1+f}=\frac{1-l_{CI}}{1+f}$.
