# Supplementary material for: Wolbachia-Induced Unidirectional Cytoplasmic Incompatibility and Speciation: Mainland-Island Model
Source: PLoS One. 2007 Aug 8;2(8):e701. doi: 10.1371/journal.pone.0000701 (PMC1934337; doi:10.1371/journal.pone.0000701)
Supplement: Text S4 — Equilibrium Frequencies of infected T 1 Individuals (0.03 MB DOC) [file pone.0000701.s004.doc]

**S4 Equilibrium Frequencies of infected *T*_1_ individuals**

Let $x_{T_{1},W}$ denote the equilibrium frequency of infected individuals with genotype *T*_1_ on the island before the reproduction step. In order to derive an approximation for $x_{T_{1},W}$we assume that migrants mate only with residents. This is justified when migration is low. Further, we assume that *s* is small and neglect the effects of local selection.

Under these assumptions we can write down $x_{T_{1},W}$ as a geometric series. The first summand, *m*, stands for migrants of the *F*_0_ generation. These are all infected and have genotype *T*_1_. The second summand, *m*(1-*f*)(1-*m*)/2, denotes the infected part of migrant offspring with genotype *T*_1_. To understand this term note first that we have to consider only the matriline because *Wolbachia* is transmitted cytoplasmically. The total number of (infected) female migrants is *m*/2 and because of the infection each of these females has on average 2(1-*f*) offspring. All of these offspring carry *Wolbachia* but only half of them have genotype *T*_1_. This is because migrants are supposed to be so rare that they have to mate exclusively with residents. Finally, we have to bear in mind that there is a new generation which is reflected by the factor (1-*m*). The full series takes into account all future generations and computes to

(33) $x_{T_{1},W}\approx m\sum_{n=0}^{\infty} \left[(1-m)\frac{1-f}{2}\right]^{n}$

By using the formula for the geometric series, we get

(34) $x_{T_{1},W}\approx\frac{2m}{1+f+m-fm}$.

Linearization around *m* yields the approximation used in the results,

(35) $x_{T_{1},W}\approx\frac{2m}{1+f}$.
