# Supplementary material for: Wolbachia-Induced Unidirectional Cytoplasmic Incompatibility and Speciation: Mainland-Island Model
Source: PLoS One. 2007 Aug 8;2(8):e701. doi: 10.1371/journal.pone.0000701 (PMC1934337; doi:10.1371/journal.pone.0000701)
Supplement: Text S5 — Incomplete transmission. (0.03 MB DOC) [file pone.0000701.s005.doc]

**S5 Incomplete Transmission**

In the present study, we have so far assumed a fecundity cost on infected female hosts, *f* > 0, while the infection was perfectly transmitted, *t* = 1. In this section, we show that qualitatively similar infection dynamics are ascertained when infected females do not suffer fecundity reductions, *f* = 0, but *Wolbachia* transmission is incomplete, *t* < 1. For this scenario, we did the same analysis described in the results. In general, incomplete *Wolbachia* transmission prevents the spread of *Wolbachia* from the mainland to the island if migration is below a critical migration rate, *m_c_* (*l*_CI_, *t*, *s*). Highest critical migration rates are observed for low levels of CI. Further, the critical migration rate increases with decreasing transmission rate and with increasing selection coefficient. In the absence of local adaptation, the critical migration rate for incomplete transmission can be determined analytically (for details see [37]) and computes to

(36) $m_{c}(l_{CI},t)=\frac{4t(1+t-2\sqrt{t})}{\left(\sqrt{l_{CI}}+\sqrt{l_{CI}-4t(1-t)}\right)^{2}}$

for *l*_CI_ > 4 *t* (1 − *t*) and *t* > 0.5. Otherwise it is zero.

In the case of local adaptation on the island, the critical migration rate can not be calculated analytically. However, it is possible to derive a lower approximation applying the analysis described in a previous part of the supplementary material (S2). Thus, it holds that

(37) $m_{c}(l_{CI},t)\geq(1+s)\frac{4t\left(1+t-2\sqrt{t}-s(1-t)\right)+2\left(l_{CI}-\sqrt{l_{CI}}\sqrt{l_{CI}-4t(1-t)}\right)}{\left((1+s)\sqrt{l_{CI}}+(1-s)\sqrt{l_{CI}-4t(1-t)}\right)^{2}+16st^{2}}$

for *l*_CI_ > 4 *t* (1 − *t*) and *t* > 0.5, and *s* ≥ 0. Note that setting *s* = 0 correctly yields formula (36). The spread of *Wolbachia* onto the island can be significantly hampered by local adaptation. If e.g. *l*_CI_ = 0.4 and *t* = 0.9 then the critical migration rate is *m_c_* = 1.4% for *s* = 0 and *m_c_* = 2.7% for *s* = 1.

If migration is below the critical migration rate then unidirectional CI acts as a postzygotic isolation mechanism. This results in gene flow reduction which facilitates the maintenance of local adaptations on the island. Applying the reasoning of section S3, the gene flow reduction can be described in terms of an effective migration rate which is approximately

(38) $m_{e}\approx m\frac{2-l_{CI}-t}{2-t}$.

E.g., if *t* = 0.95 then migration is reduced by nearly 48% for *l*_CI_ = 0.5 or by more than 95% for *l*_CI_ = 1. Numerical simulations for the scenario with incomplete transmission show that the postzygotic isolation induced by *Wolbachia* selects for premating isolation if the CI level is above a threshold.

In summary, incomplete *Wolbachia* transmission acts in a similar way as fecundity reductions do. We have further analyzed how both factors influence each other. In general, the combination of both results in an increased critical migration rate. This can significantly enlarge the region where unidirectional CI selects for local adaptations and premating isolation (results not shown).
